# Book Review

**Published:** 1912-03

**Authors:** 


**Public Health Laboratory Work.** By Henry R. Kenwood, M.B., F.R.S. Edin., and W. G. Savage, M.D., B.Sc. Fifth Edition. Pp. xiii, 446. London: H. K. Lewis. 1911.—The present edition of this admirable and exhaustive text-book of laboratory methods has been brought well up to date and generally revised. It is too well known and too widely used to need any special description in this place, but we may confidently state that it is one of the most useful of laboratory handbooks, being remarkably clear and concise in style and very reliable in detail. We note that the effect of $CO_2$ on bacterial life in water is held to render aerated bottled waters safer than ordinary water from impure sources ; that the expert tea taster can very accurately estimate the purity and genuineness by its odour and taste, which fact has recently been given further value by the experiments published in the *Lancet*, shewing that the "æsthetically" excellent teas also contain a higher percentage of caffeine and a lower percentage of free tannic acid than the so-called inferior varieties. Under the heading of poisonous and edible fungi, reference is made to the capital little book published at a very low price by the Department of Agriculture and Fisheries, which others than laboratory workers would be well advised to read. With regard to air, there is a good description of Haldane's rapid method of determining $CO_2$. The bacteriological examination of water, air, milk, etc., has been written by Dr. Savage, whose researches into these matters are well known, especially in connection with the bacteriology of milk and the group of organisms named after Gaertner. The *Lancet* method of testing disinfectants is described as "simple and serviceable." The rapid appearance of successive editions of this handbook demonstrates its popularity in a practical way; the increasing imporance and complexity of the subjects with which it so adequately deals, and the care with which each edition is revised and extended, are sufficient to account for that popularity in a highly satisfactory manner.

**Landmarks and Surface Markings of the Human Body.** By L. Bathe Rawling, M.B. Fourth Edition. Pp. viii, 96. London: H. K. Lewis. 1911.—The fact that Mr. Rawling's little book has reached its fourth edition shows that it has met with the success it deserves. Its chief merit lies in the excellence of the illustrations, which are both clear and accurate. It would be well if this type of anatomy took a much more prominent place in the student's curriculum.

**Diseases of the Spinal Cord.** By R. T. Williamson, M.D., F.R.C.P. (Second impression.) Pp. xi, 432, vii. London: Oxford Medical Publications. 1911.—It is a comparatively short time since we noticed the first edition of this authoritative work. The favourable opinion then formed of it has been endorsed by general experience to such an extent that the first impression has been exhausted and a second already issued. There are no material alterations; the book was too good to require any great overhauling and remodelling; but room has been made to add short accounts of the most important contributions to our knowledge of diseases of the spinal cord in an appendix. Reference is made to the epidemiology of infantile paralysis, to the Wassermann reaction in spinal syphilis, to spastic paraplegia of infancy (Little's disease), to the X-ray treatment of syringomyelia, and to some surgical procedures for spinal cord diseases.

**An Introduction to Dermatology.** By Norman Walker, M.D., F.R.C.P. Fifth Edition. Pp. xviii, 346. Edinburgh: William Green & Sons. 1911.—When we reviewed the fourth edition of Dr. Walker's book in 1908 we had nothing but praise for it. The fifth edition confirms us in our opinion that this is by far the best elementary work on skin disease both for students and practitioners.

**Hygiene and Public Health.** By Sir Arthur Whitelegge, K.C.B., M.D., etc., and Sir George Newman, M.D., D.P.H. Twelfth Edition. Pp. x, 760. London: Cassell & Company, Limited. 1911.—A twelfth edition needs no commendation and gives little scope for criticism. The book is intended for the health officer as well as for the student. It should be a *vade-mecum* and a mine of wealth to both. This edition has been revised throughout, and some considerable enlargement has been unavoidable since the last of three years ago. We take the opportunity of tendering our congratulations to the authors on their well-earned distinctions.

**Feeble-mindedness in Children of School Age.** By C. Paget Lapage, M.D., M.R.C.P. With an Appendix on "Treatment and Training," by Mary Dendy, M.A. Pp. viii, 359. Manchester: The University Press. 1911—This book, as the author states, is intended as a guide for school medical officers, teachers, and those social workers whose interest in the welfare of feeble-minded children has been stimulated by the Report of the recent Royal Commission on the Care and Control of the Feeble-minded. Its further objects are to emphasise the importance of public control of the feeble-minded, and the value of their permanent segregation as a means of preventing them from propagating their species. Not only does Dr. Lapage develop these points successfully, but he devotes several chapters to the medical aspect of the various types of feeble-mindedness. A valuable appendix by Miss Dendy, whose work as Honorary Secretary of the Lancashire and Cheshire Society for the Permanent Care of the Feeble-minded enables her to speak with authority on the subject, deals with the training and management of feeble-minded children, and is written on the assumption that the children to be cared for are to be detained for the whole of their lives. Altogether the book is a most excellent one.

**Where the World Ends.** By A. A. H. Pp. 95. Junginger-Hefti, Arosa, Switzerland. 1911.—The author, a former chaplain, with the assistance of Dr. Egbert C. Morland and others, has written a description of Arosa as a centre for summer holidays or winter sports and as a health resort for convalescents and invalids. To us the principal interest of Arosa is derived from the fact that it is one of the latest, the highest, and probably the best resort for early cases of consumption, those who may reasonably expect a radical cure. Both for diagnosis and treatment every facility is found: all the various bacteriological and other test methods are in full use, and X-ray examinations are done in the best way. The title indicates the position of Arosa at the far end of one of the most beautiful valleys in Switzerland, 6,000 feet above sea level, but surrounded by peaks five to six thousand feet higher. It is interesting to find in the centre of the old churchyard, Inner Arosa, the tombstone of Dr. Reudi, for many years the leading physician at Davos-Platz, who after wanderings in the Western Hemisphere ended his days amongst the mountains where he had lived. The hotels and sanatoria enable patients to do the best thing possible for their cure.

**Theory and Practice of Thyroid Therapy.** By Herbert Ewan Waller. Pp. xii, 154. London: John Bale, Sons and Danielsson, Ltd. [n.d.]—The author of this book is an enthusiast on the subject of the thyroid gland. He finds thyroid insufficiency everywhere. He considers it the cause of enlarged tonsils and adenoids, dental caries, constipation, urticaria, anæmia, rickets, nocturnal enuresis, and many other affections too numerous to mention ; accordingly he advises the administration of thyroid extract in all these and even in Graves's disease. He gives us some interesting theories about the function of the thyroid gland, mostly based on his therapeutical experience, and deprecates all operations on the thyroid gland itself. The book is well worth reading, as it is full of interesting suggestions ; but as it is not based on physiological experiment, we fear it will not bring us any nearer to an explanation of the mystery which still surrounds its subject.

**An Introduction to Therapeutic Inoculation.** By D. W. Carmalt Jones, M.A., M.D. Pp. xiii, 165. London: Macmillan & Co. Ltd. 1911.—In this book, illustrated by one coloured and four folding plates, the author has set forth in a readable manner the principles underlying vaccine therapy and the results obtained in practice. Although the work is obviously that of an enthusiast, it is well worth perusal.

**Carbonic-Acid Snow as a Therapeutic Agent in the Treatment of Diseases of the Skin.** By R. Cranston Low, M.B., F.R.C.P. Pp. xi, 117. Edinburgh: William Green & Sons. 1911.—If the first case to be treated by solid $CO_2$ snow was not until 1905, it cannot be claimed that a long experience has justified the use of this agent. On the other hand, it has abundantly proved its usefulness in this short period, and Dr. Cranston Low, in his collected notes and observations on this subject, indicates a widening field for it. Like most early publications on any new therapeutic agent, these notes of Dr. Low's are more by way of being suggestions as to methods of using the $CO_2$ snow, and some indications as to what class of cases it is likely to benefit, rather than an exhaustive monograph on the subject. Superficial lesions can undoubtedly be dealt with very effectively, and the effect upon cavernous nævi is a very happy one. If such complaints as xeroderma pigmentosum and chloasma can successfully be treated by this method it will prove of peculiar service to dermatologists. We think it necessary to enforce the warning that $CO_2$ snow produces scarring, and that there is a possibility of leaving a more extensive, if less unsightly, condition than the original lesion. We think also that there is evidence of idiosyncrasy in some persons apart from any sensitisation by means of the X-rays. We agree with Dr. Low's statement as to the treatment not being painless, for in certain parts—*e.g.* near the exits and along the course of sensory nerves, as well as round the finger-nails—the pain may be considerable. But even so there are so many ways in which $CO_2$ snow can be useful, and the apparatus is so simple to manage, and so dortable, that we believe its use will become extensive, and this work of Dr. Cranston Low's should prove a useful guide to any who care to give it a trial.

**Ambulance Examination Questions.** By D. M. MACDONALD, M.D. Pp. 24. Bristol: John Wright & Sons Ltd. 1911.—If ambulance students can answer all these questions, and use the knowledge they imply in a practical manner, they ought to be able to render "first-aid" skilfully in any emergency. A series of problems and answers are included, and a description of Prof. Schäfer's Method of Restoring Animation in the apparently drowned.

**E. Merck's Annual Report.** Vol. XXIV. Darmstadt. 1911.—This well-known annual is much appreciated, and is recognised as a reliable book of reference for the impartiality which characterises its compilation. It comprises 380 pages, including a communication of 38 pages on the therapeutic uses of the cacodylates. Medical men can obtain a free copy on application to the London office, 16 Jewry Street, E.C.

**Manual of Physiology.** By H. WILLOUGHBY LYLE, M.D., B.S., F.R.C.S. Pp. xv, 747. London: Oxford Medical Publications. 1911.—The author of this text-book has been a well-known and successful teacher of physiology at King's College, with a special reputation in preparing for the first F.R.C.S. He has now been prevailed upon to commit his teachings to paper, and a very valuable book is the result. It is smaller than the manuals in common use, but contains a good deal more information. This has been accomplished by adopting a synoptic style, omitting all reference to histology, embryology and practical methods, and curtailing the illustrations, which are mostly of a simple and diagrammatic character. The beginner would probably find it difficult to read, but for the advanced student and for purposes of reference it will be indispensable. The chapters on physiological chemistry, the eye, the structure of the central nervous system, and circulation are particularly good. Perhaps there might be a little more elaboration of the descriptions of the ductless glands, especially the pituitary, and of the functions of the semicircular canals and the cerebral cortex in the light of recent researches. Nevertheless, there is not a text-book in the English language which gives so much accurate and up-to-date information on all branches of physiology.

**A Manual of Physics for Students of Medicine.** By HUGH CANDY, B.A., B.Sc., F.I.C. Pp. viii, 384. London: Cassell and Company Ltd. 1911.—Students preparing for the examinations of the Conjoint Board, who do not wish to burden their minds with unnecessary facts, will welcome this little book, which is clearly written, copiously illustrated, and contains just enough and no more. It deals with heat, light, sound and electricity; there is a practical section at the end. It is specially written for medical students.

